# Supplementary material for: Factors influencing the effectiveness of nature-based interventions (NBIs) aimed at improving mental health and wellbeing: Protocol of an umbrella review
Source: PLoS One. 2023 Jul 21;18(7):e0273139. doi: 10.1371/journal.pone.0273139 (PMC10361498; doi:10.1371/journal.pone.0273139)
Supplement: Attachment — Submitted filename: PLOS ONE Review.docx [file pone.0273139.s003.docx]

**COMMENTS BY REVIEWER**

1. Consider extending the searches beyond May 2022
2. Aim of the umbrella review lacks clarity in lines 153-155-Revise
3. The overall objective in lines 171-175 is convoluted and confusing to the reader as it includes the aim. It also does not speak to the aim outlined in lines 153-155-Revise
4. Specify the methodology being referred to in line 190-191
5. Definition of “umbrella review” in lines 193-195 should come earlier in the introduction section
6. The following statement in line 203 sounds incomplete: “No date limit will be placed on the search until.”
7. Justify why search will be limited to peer-reviewed studies published in English only-lines 230-231-This will make you leave out articles written in other languages
8. Justify why unpublished grey literature will not be included in the review
9. Definition of NBIs presented in lines 271-273 should come earlier in the introduction section
10. Avoid unnecessary repetitions e.g lines 275-279 are a repletion of lines 71-75
11. Consider utilizing a PRIMA flow diagram as part of Methods to indicate who the selection of studies will be done
12. Provide adequate details about how statistical and clinical heterogeneity will be determined and how this will affect data analysis
13. Manuscript will benefit from grammar and language editing
