# Supplementary material for: Factors influencing the effectiveness of nature-based interventions (NBIs) aimed at improving mental health and wellbeing: Protocol of an umbrella review
Source: PLoS One. 2023 Jul 21;18(7):e0273139. doi: 10.1371/journal.pone.0273139 (PMC10361498; doi:10.1371/journal.pone.0273139)
Supplement: Attachment — Submitted filename: Response to Reviewers.docx [file pone.0273139.s004.docx]

**Response to Editor and Reviewers Comments**

Dear Editor,

We would like to extend our sincerest thanks to you and the reviewers for taking the time to evaluate our manuscript and for allowing us to revise our study. We are now submitting our revised manuscript with tracked changes and a point-by-point response to the editor and both reviewers, which can be found below.

**Academic Editor’s comments:**

1. Please ensure that your manuscript meets PLOS ONE's style requirements, including those for file naming. <https://journals.plos.org/plosone/s/file?id=wjVg/PLOSOne_formatting_sample_main_body.pdf> and

<https://journals.plos.org/plosone/s/file?id=ba62/PLOSOne_formatting_sample_title_authors_affiliations.pdf>

Thank you to the editors for this guidance. We have formatted our manuscript in line with PLOS ONE’s style guidelines.

2. During our internal evaluation of the manuscript, we found significant text overlap throughout the whole manuscript between your submission and previous work .

We would like to make you aware that copying extracts from previous publications, especially outside the methods section, word-for-word, is unacceptable. In addition, the reproduction of text from published reports has implications for the copyright that may apply to the publications.

Please revise the manuscript to rephrase the duplicated text, cite your sources, and provide details as to how the current manuscript advances on previous work. Please note that further consideration is dependent on the submission of a manuscript that addresses these concerns about the overlap in text with published work.

We will carefully review your manuscript upon resubmission and further consideration of the manuscript is dependent on the text overlap being addressed in full. Please ensure that your revision is thorough as failure to address the concerns to our satisfaction may result in your submission not being considered further.

Thank you for bringing our attention to this. Academic integrity is central to our practice, and we would never knowingly take from others work. We have addressed this concern by using Turnitin and Grammarly software to detect any text overlap. All sources have been correctly cited and we provide clear reference to how the current manuscript advances on previous work. In our check, we found that most of the overlapping was with the preprint the submitted manuscript which has been published with medrxiv: <https://www.medrxiv.org/>. No further considerable overlaps were identified by us. Please do not hesitate to let us know if further issues with originality arise.

3. We note that you have stated that you will provide repository information for your data at acceptance. Should your manuscript be accepted for publication, we will hold it until you provide the relevant accession numbers or DOIs necessary to access your data. If you wish to make changes to your Data Availability statement, please describe these changes in your cover letter and we will update your Data Availability statement to reflect the information you provide.

Thank you for this point. This is a protocol for a systematic umbrella review, thus no empirical data has been collected to prepare this manuscript. We apologize for any errors in the Data availability statement. A note about this has been made in the cover letter.

**Reviewer #1**

Thank you for the read on an important topic. I am currently teaching social work students and many of them are interested in this! Overall good language and written with clearly in-depth knowledge and passion for the topic.

I would suggest the following as improvements before publishing:

My major overall concern while reading is that the scope of the intended review is very wide. To get an overview of every intervention and try to compare them seems almost unfeasible. While I applaud the obvious advantages of trying to cover both qualitative and quantitative papers, it may in fact be better if you were to split this into two separate sister papers, "What do we know about NBI, part I" and II. If you intend to integrate both qualitative and quantitative data into all the 5 parts of the analysis I fear the result will be unwieldy. Alternatively the aims outlined ca. line 172 could be cut to 2 or 3, not 5. Without this I fear the protocol will not be replicable.

We would like to extend thanks to R1 for proposing the idea of two separate sister papers - separated into quantitative and qualitative evidence. We recognise your concerns regarding the scope of our review and objectives. We see significant merit in this idea provided the evidence would support such a divide. We have currently completed full-text screening and we are in the process of developing and piloting our data extraction sheet. By piloting the extraction sheet we aim to gauge if there is scope to divide the review into quantitative and qualitative evidence i.e. are there enough quantitative and qualitative reviews to warrant this and do the mixed-reviews present their findings in a way that would facilitate the separation of quantitative and qualitative evidence. We have added this stipulation to the protocol. See lines 620-628, p.19. Additionally we have addressed your concerns about the scope of the review being too wide by integrating objectives 3 and 4 into one (what are the factors that drive or limit the effectiveness of NBIs), and removing objective 5 but keep it as the ultimate goal of our review. See lines 281-289, p. 9

INTRO

Thorough but maybe a bit disorganised -could be written with a more clear narrative and also use subheadings if allowed by Plos. For instance, small changes like a paragraph change in line 153 would help greatly on readability.

We have organised the introduction to provide a more clear narrative of our study. Additionally, we have incorporated suggestions such as adding subheadings and paragraph changes. See introduction.

In line 160 you probably do not mean "alternatively" but maybe "in contrast" or something along those lines.

This has been amended. See line 249.

Readability could also be improved by shortcutting phrases like "It is anticipated that the qualitative analysis will capture..." -you could instead write "Our qualitative analysis will attempt to capture" -in general, avoid the passive form throughout. The sentence in question is in general hard to follow and could probably be split in two or three.

Look for potential improvements like the above throughout.

This particular example has been edited (See line 249-250) and we have altered similar instances throughout the manuscript where the passive form is used.

In addition to the organisational points I am also missing a critical voice -there could be other reasons NBI are trending right now, e.g. that it is indeed a trend to be "natural", and that the increased interest does not in fact reflect actual effect. It would be good if the intro acknowledged this and made explicit an intent to include papers overtly critical to the concept of nature based interventions. And I would like to see that you are aware that such papers may to be missing from the reviews you are reviewing and that you have considered what to do if this is the case.

I would also argue that the rise seen in interest in NBI may be partially economically motivated, and a part of the realisation on the part of western welfare states that institutions are very expensive to run, while self-help walking groups are much cheaper. In other words, I would wish for the intro to include a brief yet more nuanced and perhaps wider metaperspective on the rise of NBIs.

Thank you for this suggestion. We have provided a nuanced metaperspective on the rise of NBIs as suggested. We have also provided a more critical perspective on NBIs. See lines 142-174, p.5.

METHODS

Prisma and Prospero -good!

The "Data collection and verification" section of the paper could be much improved by providing a visual representation (table) of the extracted data plan you outline.

We have provided a visual representation (Table 1, p. 15-16) to represent our data extraction plan. See lines

You say "Exact methods of synthesis for the included qualitative research will depend on the nature of the evidence identified" - in my opinion this would need to be refined quite a bit before publishing as it is too vague. Especially with regards to replicability, this would be essential in case someone else wants to use your methods section as a blueprint for a similar project.

We have refined this statement to provide more detail on our methods of synthesis for the included qualitative studies. See lines 566-568, p. 18.

Ethics: I think whenever you are going to be investigating qualitative data, it would be pertinent to include your own pre-existing potential biases, especially given that you are most likely planning to do a narrative review.

Thank you for raising this issue. We have added a section outlining our commitment to practice reflexivity throughout the study. We will critically examine our own role, assumptions, beliefs, pre-existing potential bias and impact on the data during all stages of the research process including: (a) formulation of the research questions (b) data collection and (c ) data analysis. We hope that this reflexive engagement by multiple analysts will enhance the quality of this research. See lines 612-618, p.19.

**Reviewer #2**

Thank you R2 for your helpful and constructive feedback. Below we outline our response to your comments and subsequent action taken.

1. Consider extending the searches beyond May 2022

The search will be extended beyond May 2022. No date limit will be placed on the search. See line 322, p.10.

2. Aim of the umbrella review lacks clarity in lines 153-155-Revise

We have clarified our aim by adding more specific detail to this explanation. See lines 189-194.

3. The overall objective in lines 171-175 is convoluted and confusing to the reader as it includes the aim. It also does not speak to the aim outlined in lines 153-155-Revise

We have edited this to make sure that our overall objective links to the aim mentioned earlier in the introduction. Additionally, we have integrated objectives 3 and 4 into one (what are the factors that drive or limit the effectiveness of NBIs), and removed objective 5 but keep it as the ultimate goal of our review. We hope that this has provided a more concise overview of our specific objectives. See lines 237—289, p.9.

4. Specify the methodology being referred to in line 190-191

The specific methodology we were referring to has been outlined. See lines 246-247.

5. Definition of “umbrella review” in lines 193-195 should come earlier in the introduction section

Definition has been moved to earlier in the introduction section. See lines 200-202, p.7.

6. The following statement in line 203 sounds incomplete: “No date limit will be placed on the search until.”

This has been corrected. See line 322.

7. Justify why search will be limited to peer-reviewed studies published in English only-lines 230-231-This will make you leave out articles written in other languages

Our decision to include only peer-reviewed studies is based on our commitment to provide a synthesis of high quality evidence which has gone through a meticulous and rigorous review process. Our decision to include only studies published in English is the result of limited resources and the language constraints of our review team. While we acknowledge that this is a limitation of our review, including studies published in non-English languages would have increased resource challenges in relation to time, costs, and expertise in non-English languages. We have clarified this in the manuscript. See lines 357-363, p.11.

8. Justify why unpublished grey literature will not be included in the review

This decision is linked to our aforementioned commitment to include only studies which have gone through a rigorous review process which impacts upon study quality. This has been clarified (Aromataris et al., 2015) <https://journals.lww.com/ijebh/Fulltext/2015/09000/Summarizing_systematic_reviews__methodological.4.aspx> See lines 357-363.

9. Definition of NBIs presented in lines 271-273 should come earlier in the introduction section

This definition is now in the introduction section. See lines 311-312.

10. Avoid unnecessary repetitions e.g lines 275-279 are a repletion of lines 71-75

This repetition has been removed.

11. Consider utilizing a PRIMA flow diagram as part of Methods to indicate who the selection of studies will be done

We thank the Reviewer for this suggestion. We have made more evident our approach to data selection at p.12, where we have clarified the following:

“A PRISMA flow chart will be developed to record the screening and selection of studies. Once all records from our search are collected, EndNote 20 software (Thomson Reuters, Toronto, Ontario, Canada) will be used to remove duplicates and screen literature. A 2-stage screening process will be completed independently by two researchers (TS and CVYC); the first screening stage will consider titles and abstracts, while full-texts will be checked in the second stage”

A completed PRISMA flow diagram has not been included in the protocol as we would not be able to populate it until screening is completed.

12. Provide adequate details about how statistical and clinical heterogeneity will be determined and how this will affect data analysis

We have clarified in the Data Analysis section (p.18, line 556-560) that:

“Where possible and appropriate, statistical heterogeneity will be assessed following the Crochane 6.3 guidance (Deeks et al., 2022). Should trials of clinical relevance be included, an assessment of clinical heterogeneity, considering variability in participants, outcomes and characteristics of the intervention, will be assessed following guidance by Gagnier et al. (2013).”

13. Manuscript will benefit from grammar and language editing

Our review team has assessed the text for any grammar or language issues. Additionally, we have used specific software i.e. Grammarly to make changes to our grammar and language throughout the manuscript.
