# Supplementary material for: Factors influencing the effectiveness of nature-based interventions (NBIs) aimed at improving mental health and wellbeing: Protocol of an umbrella review
Source: PLoS One. 2023 Jul 21;18(7):e0273139. doi: 10.1371/journal.pone.0273139 (PMC10361498; doi:10.1371/journal.pone.0273139)
Supplement: Attachment — Submitted filename: Manuscript Number PONE.docx [file pone.0273139.s005.docx]

**Manuscript Number PONE-D-22-21700**

**"Factors influencing the effectiveness of nature–based Interventions (NBIs) aimed at improving mental health and wellbeing: Protocol of an umbrella review"**

1. **References : some of the references are outdated . authors can chose the recent 5 years papers.**
2. **Authors should simply the text and focus on grammar more.**
